# Retinal microvascular parameters are not significantly associated with mild cognitive impairment in the Northern Ireland Cohort for the Longitudinal Study of Ageing

**DOI:** 10.1186/s12883-021-02137-4

**Published:** 2021-03-11

**Authors:** R. A. O’Neill, A. P. Maxwell, E. N. Paterson, F. Kee, I. Young, R. E. Hogg, S. Cruise, S. Murphy, B. McGuinness, G. J. McKay

**Affiliations:** Centre for Public Health, Queens University Belfast, Belfast, Northern Ireland

**Keywords:** Retinal microvascular parameters, Cognitive function, Mild cognitive impairment, The Montreal cognitive assessment

## Abstract

**Background:**

The retinal and cerebral microvasculature share similar embryological origins and physiological characteristics. Improved imaging technologies provide opportunistic non-invasive assessment of retinal microvascular parameters (RMPs) against cognitive outcomes. We evaluated baseline measures for associations between RMPs and mild cognitive impairment (MCI) from participants of the Northern Ireland Cohort for the Longitudinal Study of Ageing (NICOLA).

**Methods:**

RMPs (central retinal arteriolar / venular equivalents, arteriole to venular ratio, fractal dimension and tortuosity) were measured from optic disc centred fundus images and analysed using semi-automated software. Associations between RMPs and MCI were assessed by multivariable logistic regression with adjustment for potential confounders including age, sex, alcohol consumption, smoking status, educational attainment, physical activity, cardiovascular disease (CVD), hypertension, mean arterial blood pressure, triglycerides, diabetes, body mass index, and high density lipoprotein levels. *P* < 0.05 was considered statistically significant.

**Results:**

Data were available for 1431 participants, of which 156 (10.9%) were classified with MCI defined by a Montreal Cognitive Assessment (MoCA) score ≤ 26, with subjective cognitive decline, in the absence of depression or problems with activities of daily living. Participants had a mean age of 62.4 ± 8.5 yrs. and 52% were female. As expected, individuals with MCI had a lower MoCA score than those without (23.5 ± 2.6 versus 26.3 ± 2.7, respectively), were more likely to be female, have a lower level of educational attainment, be less physically active, more likely to have CVD, have higher levels of triglycerides and lower levels of high density lipoprotein. No significant associations between RMPs and MCI were detected in unadjusted, minimally adjusted or fully adjusted regression models or subsequent sensitivity analyses.

**Conclusion:**

Previous studies have reported both increased retinal venular calibre and reduced fractal dimension in association with mild cognitive impairment. Our study failed to detect any associations between RMPs and those individuals at an early stage of cognitive loss in an older community-based cohort.

**Supplementary Information:**

The online version contains supplementary material available at 10.1186/s12883-021-02137-4.

## Background

Population ageing has resulted in increased prevalence of age-related conditions that negatively impact healthcare systems, policies and demands, primarily as a consequence of reduced mortality [1, 2]. The most rapidly growing demographic in Northern Ireland (NI) is those aged > 50 years (yrs), necessitating improved understanding of the impact of age-related conditions [3]. Cognitive impairment significantly impacts activities of daily living (ADL), especially among the aged [4, 5]. During the ageing process, cognitive skills such as conceptual reasoning, memory, and processing speed decline. Some individuals may exceed a threshold characterised as mild cognitive impairment (MCI), which may progress to dementia in up to 50% of cases over 5 years [6–11]. MCI is characterised by subtle changes in cognitive function, although everyday life generally remains largely unaffected. Severe cognitive impairment that leads to dementia results in an extensive loss of cognitive ability and ultimately, independent living [12]. Given life expectancy has increased globally, an improved understanding of normal ageing processes will advance measures of successful and healthy ageing and differentiation of normal and diseased states [13, 14].

The characteristic disease aetiology of MCI is not fully understood, despite its influence on increased morbidity among the elderly. Cerebral microvascular disease has previously been associated with an increased risk of both MCI and Alzheimer’s disease [15–19], possibly through common microvascular factors such as hypertension, diabetes, smoking and inflammation [20–22]. Furthermore, variation in cerebral microvascular characteristics, such as increased tortuosity and arteriolar narrowing, have been reported in association with degenerative changes during the onset of dementia [23, 24]. The retinal and cerebral microvasculature share similar embryological origins and physiological characteristics [25], and advanced retinal imaging has facilitated the non-invasive assessment of associations between retinal microvascular parameters (RMPs) and cognitive function [25–29]. Previously, reduced retinal microvascular calibre and fractal dimension have been reported in association with cognitive impairment with suggestions that this may reflect cerebral microvascular variation, especially in older adults [30, 31]. The aim of this study was to evaluate associations between RMPs and MCI in the Northern Ireland Cohort for the Longitudinal Study of Ageing (NICOLA).

## Methods

### Study population

NICOLA is a longitudinal cohort study of 8468 participants aged 50 years and over, living in the Northern Ireland community (individuals in care homes or other residential institutions were excluded [32, 33];). The study was established in 2012 with three main components: a computer-aided personal interview (CAPI), a self-completion questionnaire and health assessment. The CAPI extensively assessed demographic, social and health-related factors. Biological samples and measures of cardiovascular, physical, cognitive and visual function, including retinal fundus photography, were collected. Ethical approval was provided by the School of Medicine, Dentistry and Biomedical Sciences Ethics Committee, Queen’s University Belfast and written informed consent obtained prior to participation, in accordance with the Declaration of Helsinki (SREC 12/23).

### Measurement of cognitive function and classification of MCI

Data was available for 3741 participants that attended a health assessment which included a 30 point Montreal Cognitive Assessment (MoCA) to evaluate cognitive function. This included a short-term memory recall task, assessment of visuospatial abilities and executive function, a phonemic fluency task, and a two-item verbal abstraction task. Attention, concentration, language, orientation to time and working memory were also assessed [13]. An extra point was added to the MoCA test score for participants with less than 12 years of formal education. Participants were investigated for subjective cognitive decline (SCD) and a series of questions based on difficulties associated with basic ADL, such as dressing, walking, bathing or showering, eating, getting in or out of bed, and using the toilet. SCD was assessed qualitatively at CAPI by participant response to a five point rating of their day-to-day memory with fair to poor, the least favourable options, characterised as SCD. Participants also completed the Centre for Epidemiologic Studies Depression Scale questionnaire (CES-D) which consisted of 20 questions that were scored from 0 to 3, to assess depressive symptoms. Summative scores ranged from 0 to 60, with values ≥16 indicative of depression (DEPR [34];). Individuals who failed to complete the MoCA, reported difficulties with ADL activities, and had DEPR (CES-D ≥ 16) or retinal images of insufficient quality, were excluded from the analysis. MCI was defined as a combination of MoCA test score ≤ 26 with SCD, in the absence of DEPR or problems with ADL activities. In a sensitivity analysis, a stricter MCI definition used a MoCA score ≤ 23 in the presence of SCD or problems with ADL activities and the absence of DEPR.

### Other variables

Mean arterial blood pressure (MABP), was taken as the average of two individual systolic blood pressure (SBP) and diastolic blood pressure (DBP) measurements (2/3 DBP + 1/3 SBP). Diabetic status was defined using a combination of participant percentage haemoglobin A1c (HbA1c > 6.5%), diabetic medication use or self-reported diabetes. Cardiovascular disease (CVD) was by self-report and included a history of angina, heart attack, congestive heart failure or stroke. Educational attainment was dichotomised on the basis of primary or secondary level education and above (including university education). Smoking status was dichotomised as current versus non-smoker. Alcohol consumption was categorised into four groups; non-drinker, light drinker (0–7 units per week), moderate drinker (7–14 units per week) and heavy drinker (> 14 units per week). Physical activity (PA) was categorised as low, moderate or highly active in accordance with the Global Physical Activity Questionnaire (GPAQ) by calculating the average time per day spent in each activity domain (work, transport and leisure) and the intensity of that time spent [35, 36]. Hypertension was categorised on the basis of self-report or use of antihypertensive medication.

### Measurement of retinal images

**Fig. 1:**
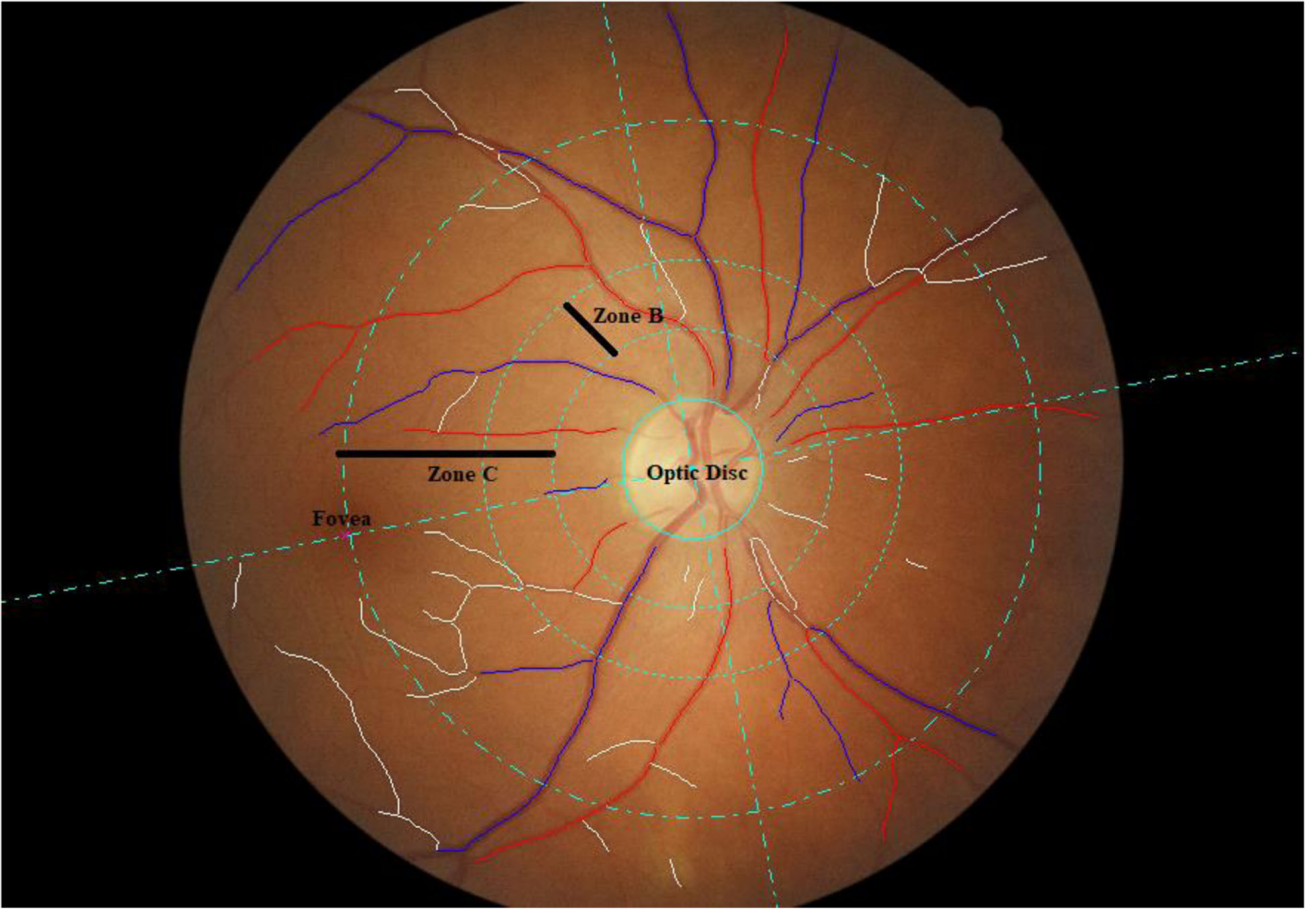
Retinal fundus image assessment using VAMPIRE software. Optic disc centred retinal fundus image assessment using the Vessel Assessment and Measurement Platform for Images of the Retina (VAMPIRE) software. Arterioles (red), venules (blue) and deleted segments (white) are indicated. The retinal microvascular parameters for arteriolar and venular calibre (CRAE, CRVE, and AVR), are calculated from measurements captured in zones B (1.0 to 1.5 optic disc diameters from the centre of the optic disc). Fractal dimension and tortuosity are calculated from measurements captured in zone C (1.0 to 2.5 optic disc diameters from the centre of the optic disc)

Retinal photography was undertaken following dilation from a single drop of 1% tropicamide using a Canon CX-1 Digital Fundus Camera (Canon USA, Melville, NY, USA). RMPs included central retinal arteriolar equivalent (CRAE), central retinal venular equivalents (CRVE), arteriole to venular ratio (AVR), fractal dimension and tortuosity quantified from optic disc centred fundus images using the semi-automated software Vessel Assessment and Measurement Platform for Images of the Retina (VAMPIRE; VAMPIRE group, University of Dundee, Dundee, Scotland, Version 3.1, Fig. 1), by a trained grader that was blinded to participant data [37, 38]. Analysis was performed mostly on left eye retinal images except when unavailable or of insufficient quality, in which case right eye images was used. A paired samples t-test was used to compare a sub-sample of left and right eye measures from 75 participants. Intraclass correlation coefficients (ICCs) were compared to determine intergrader reliability with mean values of 0.87 (CRAE) and 0.91 (CRVE).

### Statistical analysis

All statistical tests were completed using Statistical Package for Social Sciences (Version 24.0. Armonk, NY: IBM Corp). All RMPs were transformed into standardised Z-scores (a standard deviation [SD] increase or decrease from the mean) before consideration within regression models. Population summary measures were described using mean and SD for continuous variables or frequencies and percentages for categorical variables. Independent samples t-tests and chi-squared tests compared the distribution of participant characteristics with and without MCI. Logistic regression evaluated associations between RMPs and MCI status categorised as a binary trait. Minimally adjusted models included age and sex, while fully adjusted models also included alcohol consumption, smoking status, educational attainment, PA, CVD, hypertension, MABP, triglycerides, diabetes, body mass index (BMI) and high density lipoprotein (HDL). *P* < 0.05 was considered statistically significant.

## Results

**Fig. 2:**
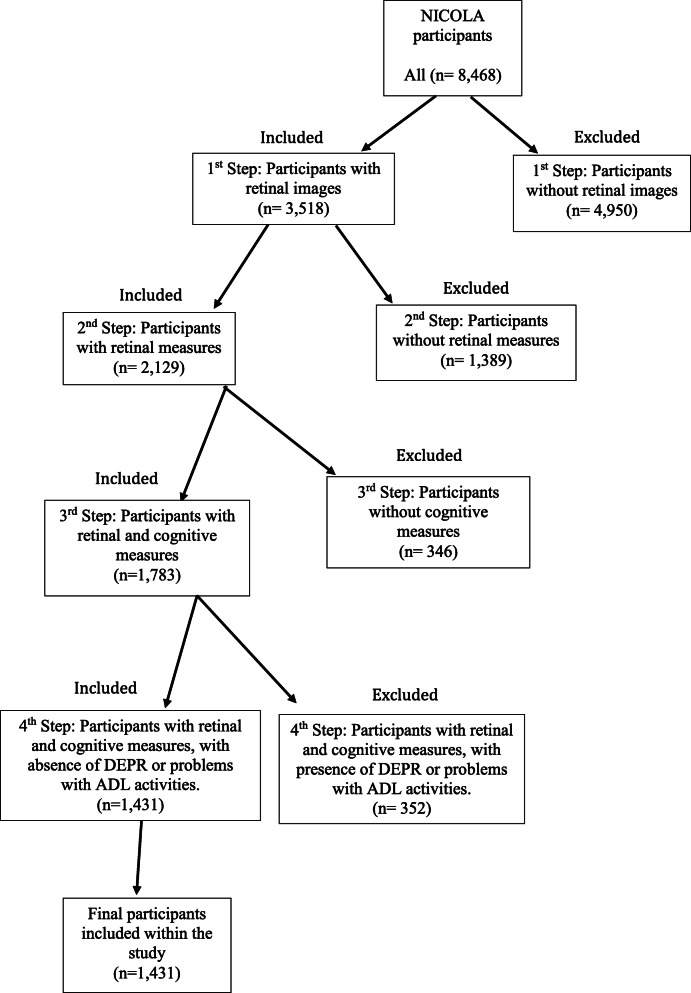
A flow chart of participant inclusion and exclusion criteria

Data were available for 1431 participants who met the study inclusion criteria (Fig. 2). There were 156 (10.9%) and 1275 (89.1%) participants classified with and without MCI respectively. Participants had a mean age of 62.4 ± 8.5 yrs. and 52% were female, with 87% categorised with an educational attainment of secondary level or above (including higher education and university; Table 1). The mean MoCA score for all participants was 26.0 ± 2.8. MABP was 98.1 ± 12.6 mmHg, and 22, 6 and 30% of participants were characterised with diabetes, CVD and hypertension, respectively. Individuals with MCI had a lower MoCA score than those without (23.5 ± 2.6 versus 26.3 ± 2.7, respectively), had a higher mean age (64.4 ± 8.8 yrs. versus 62.1 ± 8.4 yrs) and a lower percentage were female (45% versus 53%, respectively). Those with MCI had higher mean triglycerides (1.8 ± 1.2 mmol/L versus 1.6 ± 0.9 mmol/L, respectively) and evidence of CVD (12% versus 6%). Those without MCI had higher mean HDL cholesterol (1.7 ± 0.5 mmol/L versus 1.6 ± 0.4 mmol/L, respectively) and educational attainment (89% versus 76%, Table 1).

**Table 1:** Participant summary characteristics

Patient characteristics	All (***n*** = 1431)	No MCI (***n*** = 1275)	MCI (***n*** = 156)	***P***-Value
Mean age (years, SD)	62.4 ± 8.5	62.1 ± 8.4	64.4 ± 8.8	< 0.01
Female, n (%)	750 (52.4)	680 (53.3)	70 (44.9)	0.05
Smoking status, yes n (%)	115 (8.0)	101 (7.9)	14 (9.0)	0.65
Alcohol consumption, non-drinker, n (%)	299 (20.9)	261 (20.5)	38 (24.4)	0.63
Education, secondary level and above, n (%)	1251 (87.4)	1133 (88.9)	118 (75.6)	< 0.01
Physical activity level, highly active, n (%)	438 (30.6)	405 (31.8)	33 (21.2)	0.04
Diabetes, yes n (%)	308 (21.5)	275 (21.6)	33,921.2)	0.91
Mean BMI (kg/m^2^, SD)	28.2 ± 4.6	28.1 ± 4.6	28.9 ± 4.5	0.03
Mean arterial blood pressure (mmHg, SD)	98.1 ± 12.6	98.2 ± 12.6	97.1 ± 12.5	0.30
Cardiovascular disease, yes n (%)	90 (6.3)	72 (5.6)	18 (11.5)	< 0.01
Hypertension, yes n (%)	432 (30.2)	372 (29.2)	60 (38.5)	0.02
Mean triglyceride (mmol/L, SD)	1.6 ± 0.9	1.6 ± 0.9	1.8 ± 1.2	0.01
Mean HDL cholesterol (mmol/L, SD)	1.6 ± 0.5	1.7 ± 0.5	1.6 ± 0.4	0.02
Mean MOCA test score (SD)	26.0 ± 2.8	26.3 ± 2.7	23.5 ± 2.6	< 0.01

Values are n (%) for categorical variables and mean ± SD for continuous variables. *P* values were calculated by independent samples t and chi squared tests. Abbreviations: *MCI* mild cognitive impairment, *BMI* body mass index, *HDL* high-density lipoprotein, *MoCA* Montreal Cognitive Assessment, *SD* standard deviation. *P* < 0.05 was considered statistically significant

Left and right eye CRAE and CRVE measures from 75 participants were not significantly different (P_Crae_ = 0.08; P_Crve_ = 0.89). No significant associations between RMPs and MCI in unadjusted (Table 2), minimally adjusted (which included age and sex) or fully adjusted comparisons (which also included alcohol consumption, smoking status, educational attainment, PA, CVD, hypertension, MABP, triglycerides, diabetes, BMI, and HDL; Table 3).

**Table 2:** Summary of participant retinal microvascular parameters

Retinal microvascular parameters	All (***n*** = 1431)	No MCI (***n*** = 1275)	MCI (***n*** = 156)	***P***-Value
Mean CRAE (PX, SD)	29.643 ± 2.221	29.632 ± 2.213	29.731 ± 2.294	0.60
Mean CRVE (PX, SD)	40.844 ± 3.268	40.858 ± 3.293	40.723 ± 3.059	0.62
Mean AVR (SD)	0.729 ± 0.061	0.728 ± 0.061	0.733 ± 0.064	0.38
Mean fractal dimension arteriolar (SD)	1.557 ± 0.053	1.557 ± 0.053	1.555 ± 0.053	0.60
Mean fractal dimension venular (SD)	1.540 ± 0.051	1.540 ± 0.050	1.539 ± 0.052	0.78
^a^Mean tortuosity arteriolar (SD)	0.114 ± 0.157	0.112 ± 0.157	0.128 ± 0.162	0.24
^a^Mean tortuosity venular (SD)	0.067 ± 0.106	0.068 ± 0.111	0.063 ± 0.056	0.61

Values are n (%) for categorical variables and mean ± SD for continuous variables. *P* values were calculated by independent samples t tests. Abbreviations: *MCI* mild cognitive impairment, *CRAE* central retinal arteriolar equivalent, *CRVE* central retinal venular equivalent, *AVR* retinal arteriole/venular ratio, *SD* standard deviation, *PX* Pixels. ^a^Tortuosity values were multiplied by 1000 in order to be shown in Table. *P* < 0.05 was considered statistically significant

**Table 3:** Logistic regression analysis of retinal microvascular parameters and Mild Cognitive Impairment status

Retinal parameter	Minimally Adjusted			Fully Adjusted		
	OR	95% CI	***P***-Value	OR	95% CI	***P***-Value
^a^CRAE (PX)	1.04	0.88, 1.23	0.66	1.04	0.88, 1.24	0.63
^a^CRVE (PX)	0.95	0.80, 1.12	0.53	0.96	0.81, 1.14	0.67
^a^AVR	1.08	0.92, 1.28	0.35	1.07	0.90, 1.28	0.42
^a^Fractal dimension arteriolar	0.98	0.83, 1.15	0.80	0.93	0.79, 1.10	0.42
^a^Fractal dimension venular	1.00	0.85, 1.18	0.98	0.97	0.82, 1.15	0.69
^ab^Tortuosity arteriolar	1.12	0.95, 1.32	0.19	1.09	0.92, 1.29	0.30
^ab^Tortuosity venular	0.97	0.82, 1.15	0.76	0.97	0.82, 1.16	0.75

Abbreviations: *CRAE* central retinal arteriolar equivalent, *CRVE* central retinal venular equivalent, *AVR* retinal arteriole/venular ratio, *CI* confidence interval, *OR* odds ratio, *PX* pixels. ^a^RMPs were transformed into standardised Z-scores before inclusion in regression models. ^b^Tortuosity values were skewed and therefore log-transformed before inclusion in regression models. Minimally adjusted model: age and sex. Fully adjusted model: age, sex, alcohol consumption, smoking status, educational attainment, physical activity, history of cardiovascular disease, hypertension, triglycerides, diabetes, medication, mean arterial blood pressure, body mass index and high density lipoprotein. *P* < 0.05 was considered statistically significant

A sensitivity analysis to examine a more severe MCI phenotype defined by a MoCA threshold ≤23 in the presence of SCD or problems with ADL activities and the absence of DEPR reclassified some of the 1431 participants characterising 61 (4.3%) and 1370 (95.7%) with and without MCI, respectively. As expected, individuals with MCI had a lower MoCA score than those without (20.8 ± 2.0 versus 26.2 ± 2.7, respectively), had a higher mean age (66.4 ± 9.4 yrs. versus 62.2 ± 8.4 yrs) and lower percentage female (43% versus 53%, respectively; Supplementary Table 1). AVR was significantly associated with MCI in unadjusted (Supplementary Table 2) and minimally adjusted models (which included age and sex; *P* = 0.04), but did not survive full adjustment for age, sex, alcohol consumption, smoking status, educational attainment, PA, CVD, hypertension, MABP, triglycerides, diabetes, BMI and HDL (*P* > 0.05; Supplementary Table 3). No further significant associations were detected between RMPs and MCI in unadjusted (Supplementary Table 2), minimally adjusted or fully adjusted comparisons (*P* > 0.05; Supplementary Table 3).

## Discussion

Advances in imaging technologies have enabled in vivo non-invasive, opportunistic, quantitative evaluation of retinal characteristics with vascular health [39]. Previous population-based studies reported associations between RMPs and MCI [15, 29, 30] although these have been mostly limited to vessel calibre with several also considering fractal dimension and tortuosity [31, 40]. A recent systematic review and meta-analysis of five studies reported associations between decreased arteriolar and venular fractal dimension and cognitive impairment [41]. The case definitions used in all five studies were variable and included a significant proportion of participants with more advanced dementia [41]. Although, we were unable to replicate their findings, the direction of effect observed in our study were similar, despite the less severe phenotype within our population-based study.

Liew and colleagues characterised 121 participants with MCI (6.1%) according to a Mini Mental State Examination (MMSE) threshold ≤23 from 1988 participants aged 49–97 yrs. from the cross-sectional population-based Blue Mountain Eye Study. They reported associations between wider venular calibre and cognitive impairment, the strongest being in persons with hypertension, and hypothesised this may reflect cerebral venular narrowing associated with cognitive decline [30]. In contrast, we found little support of association between CRVE and MCI in the current study (OR = 0.96; 95% CI: 0.81, 1.15; *P* = 0.68). Other studies have suggested a MoCA threshold of 26 may limit the specificity of cognitive assessment through an over estimation of those affected [42–46]. Interestingly, a recent meta-analysis reported improved diagnostic accuracy using a MoCA threshold ≤23 to reduce the number of false-positive MCI participants [47]. In addition, our findings also failed to support the previously reported associations between retinal arteriolar narrowing in 809 older Latinos [48]. To improve the sensitivity of the MCI definition in our study, those with DEPR were excluded and the MoCA threshold reduced ≤23 in the presence of SCD or problems with ADL activities. This sensitivity analysis also failed to detect any significant associations between RMP and MCI (Supplementary Tables 1, 2 and 3). Further analyses that used an MCI definition not constrained by the presence of DEPR (CES-D score ≥ 16), also failed to identify any significant associations in a fully adjusted binary logistic regression model that also included CES-D score as a covariate (data not shown).

Our study had several strengths including the large population-based study design. The participants were well-characterised, with a significant number of potential confounders considered, including demographic factors, clinical variables, co-morbidities and medications. Furthermore, the availability of optic disc centred retinal fundus images provided a more accurate quantification of RMPs compared to macula centred images, which often limit assessment to the retinal temporal arcades. In this study, retinal images from the left eye were analysed except when unavailable or of insufficient quality, in which case right eye images, were used. Use of data from a single eye is unlikely to have limited the study outcomes, as similar investigations have previously reported high bilateral RMP comparisons [49–51], similarly supported by data from a subset of NICOLA participants.

Characterisation of MCI (based on MoCA ≤26 and SCD with no limitations in ADL, independent of DEPR) was well defined and in line with similar studies [52]. The MoCA was better suited to our study population as it was primarily developed as a valid and reliable screening tool for MCI, in turn, offering greater sensitivity and specificity in comparison to the MMSE [13, 44, 53, 54]. Previous studies have suggested MoCA offers improved sensitivity for the characterisation of MCI, dementia, Alzheimer’s disease, stroke and Parkinson’s disease [13, 43, 44, 53, 55–58]. Exclusion of participants with DEPR further improved the accuracy of MCI characterisation, given those with depressive symptoms are more likely to score poorly in a MoCA [59]. Furthermore, differences between mild and major neurocognitive disorders are determined by the degree to which cognitive decline restricts daily functioning. In major neurocognitive disorders or dementia, cognitive impairment negatively impacts independence in basic ADLs such as walking, bathing and eating. In comparison, those with mild neurocognitive disorders or MCI, largely remain self-sufficient, although subtle problems may arise in complex activities (instrumental ADLs) [60]. Therefore, the exclusion of individuals reporting difficulties with basic ADLs strengthens the MCI definition and reduces the likelihood of including those with more advanced cognitive impairment.

The NICOLA participants were aged 50 yrs. and above, with a mean age of 62 years, thereby limiting the number of participants with MCI. In the Cardiovascular Health Study, MCI prevalence increased from 19% in participants < 75 yrs. to 29% in those 85 yrs. and older [61]. Kumar and colleagues reported lower MCI prevalence in participants aged 60 to 64 yrs. (3.7%), a demographic similar to our study [62]. Tyrovolas and colleagues, reported MCI prevalence of 10.1% in 5364 participants of the Irish Longitudinal Study on Ageing (TILDA), not dissimilar to the comparably aged NICOLA participants in the current study (10.9%) [52].

Our study had some limitations. Although NICOLA is a longitudinal study, only baseline data were available for the present analysis. The largely Caucasian study participants aged ≥50 yrs. excluded institutionalised individuals and those with dementia and may represent the ‘worried-well’, i.e. those that attended the health assessment were more likely to have been conscious about their health, thereby limiting the generalisability of our findings to the wider population. Despite adjustment for potential confounders, the possibility of residual confounding remains. Retinal imaging and cognitive function measures were only available for the 1431 participants that met the inclusion criteria which represented only 38% of those that attended for health assessment and may represent a further source of selection bias (Supplementary Table 4). It was not feasible to undertake a formal neuropsychological assessment within the NICOLA study. We determined MCI using the most robust measures of cognition (MoCA), subjective concerns (SCD), function (ADLs) and depressive symptoms (CES-D) available. Finally, MCI is not consistently characterised across all population-based studies, although our definition was comparable to similar studies [52].

## Conclusion

The increasing prevalence of MCI highlights the importance of sufficient provision within healthcare systems for the early identification of at risk individuals in this emerging public health problem. Despite previously reported associations between wider retinal venular calibre and reduced measures of retinal fractal dimension and MCI, we were unable to find any supporting evidence that variation in retinal microvascular parameters might help identify those individuals at increased risk.

## Supplementary Information

**Additional file 1: Supplementary Table 1.** Participant summary characteristics using a Montreal Cognitive Assessment score of ≤23 in the presence of SCD or problems with ADL activities and the absence of DEPR. **Supplementary Table 2.** Summary of participant retinal microvascular parameters using a Montreal Cognitive Assessment score of ≤23 in the presence of SCD or problems with ADL activities and the absence of DEPR. **Supplementary Table 3.** Logistic regression analysis of retinal microvascular parameters and Mild Cognitive Impairment status characterised by a Montreal Cognitive Assessment score of ≤23 in the presence of SCD or problems with ADL activities and the absence of DEPR. **Supplementary Table 4.** Comparison of demographic characteristics between all participants with retinal fundus imaging with and without VAMPIRE retinal measures.

## Data Availability

The data that support the findings of this study are available from the Northern Ireland Cohort for Longitudinal Ageing (NICOLA) but restrictions apply to the availability of this data. Data access is available by request through the NICOLA Data Access Committee (https://www.qub.ac.uk/sites/NICOLA/InformationforResearchers/).

## Abbreviations

NICOLA: The Northern Ireland Cohort for the Longitudinal Study of Ageing
BMI: Body mass index
HDL: High-density lipoprotein
CRAE: Central retinal arteriolar equivalent
CRVE: Central retinal venular equivalent
AVR: Retinal arteriole/venular Ratio
SD: Standard deviation
PX: Pixels
SBP: Systolic blood pressure
DBP: Diastolic blood pressure
CI: Confidence intervals
β: Beta value
ICC’s: Intraclass correlation coefficients
VAMPIRE: Vessel Assessment and Measurement Platform for Images of the Retina
MABP: Mean arterial blood pressure
RMPs: Retinal microvascular parameters
Yrs: Years
MoCA: Montreal Cognitive Assessment
MCI: Mild cognitive impairment
CVD: Cardiovascular disease
CAPI: Computer-aided personal interview
GPAQ: Global Physical Activity Questionnaire
NI: Northern Ireland
MMSE: Mini-Mental State Examination
ADL: Activities of daily living
SCD: Subjective cognitive decline
DEPR: Depression
CES-D: CENTRE for Epidemiologic Studies Depression Scale questionnaire
PA: Physical activity
