# Supplementary material for: Retinal microvascular parameters are not significantly associated with mild cognitive impairment in the Northern Ireland Cohort for the Longitudinal Study of Ageing
Source: BMC Neurol. 2021 Mar 11;21:112. doi: 10.1186/s12883-021-02137-4 (PMC7948373; doi:10.1186/s12883-021-02137-4)
Supplement: Supplementary file 1 — Additional file 1: Supplementary Table 1. Participant summary characteristics using a Montreal Cognitive Assessment score of ≤23 in the presence of SCD or problems with ADL activities and the absence of DEPR. Supplementary Table 2. Summary of participant retinal microvascular parameters using a Montreal Cognitive Assessment score of ≤23 in the presence of SCD or problems with ADL activities and the absence of DEPR. Supplementary Table 3. Logistic regression analysis of retinal microvascular parameters and Mild Cognitive Impairment status characterised by a Montreal Cognitive Assessment score of ≤23 in the presence of SCD or problems with ADL activities and the absence of DEPR. Supplementary Table 4. Comparison of demographic characteristics between all participants with retinal fundus imaging with and without VAMPIRE retinal measures. [file 12883_2021_2137_MOESM1_ESM.docx]

**Supplementary Table 1**. Participant summary characteristics using a Montreal Cognitive Assessment score of ≤ 23 in the presence of SCD or problems with ADL activities and the absence of DEPR.

| **Patient characteristics** | **All (n=1431)** | **No MCI (n=1370)** | **MCI (n=61)** | **P-Value** |
| --- | --- | --- | --- | --- |
| Mean age (years, SD) | 62.4 ± 8.5 | 62.2 ± 8.4 | 66.4 ± 9.4 | <0.01 |
| Female, n (%) | 750 (52.4) | 724 (52.8) | 26 (42.6) | 0.12 |
| ^a^Smoking status, no n (%) | 1316 (92.0) | 1260 (92.0) | 56 (91.8) | 0.96 |
| Alcohol consumption, non-drinker, n (%) | 299 (20.9) | 277 (20.2) | 22 (36.1) | 0.02 |
| Education, secondary level and above, n (%) | 1251(87.4) | 1214 (88.6) | 37 (60.7) | <0.01 |
| Physical activity level, highly active, n (%) | 438 (30.6) | 425 (31.0) | 13 (21.3) | <0.01 |
| ^a^Diabetes, no n (%) | 1123 (78.5) | 1070 (78.1) | 54 (88.5) | 0.05 |
| Mean BMI (kg/m^2^, SD) | 28.2 ± 4.6 | 28.2 ± 4.6 | 28.1 ± 4.9 | 0.93 |
| Mean arterial blood pressure (mmHg, SD) | 98.1 ± 12.6 | 98.2 ± 12.5 | 95.8 ± 12.8 | 0.15 |
| ^a^Cardiovascular disease, no n (%) | 1341 (93.7) | 1289 (94.1) | 52 (85.2) | <0.01 |
| Hypertension, yes n (%) | 432 (30.2) | 411 (30.0) | 21 (34.4) | 0.46 |
| Mean triglyceride (mmol/L, SD) | 1.6± 0.9 | 1.6 ± 0.9 | 1.7 ± 1.2 | 0.25 |
| Mean HDL cholesterol (mmol/L, SD) | 1.6 ± 0.5 | 1.6 ±0.5 | 1.5 ± 0.4 | 0.04 |
| Mean MOCA test score (SD) | 26.0 ± 2.8 | 26.2 ± 2.7 | 20.8 ± 2.0 | <0.01 |

Values are n (%) for categorical variables and mean ± SD for continuous variables. P values were calculated by independent samples t and chi squared tests. Abbreviations: MCI, mild cognitive impairment; BMI, body mass index; HDL, high-density lipoprotein; MoCA, Montreal Cognitive Assessment;; SD, standard deviation. P < 0.05 was considered statistically significant.

^a^ characteristics with inverted values compared to table 1. This was due to cell counts < 10 which would contravene the NICOLA data access policy.

**Supplementary Table 2**. Summary of participant retinal microvascular parameters using a Montreal Cognitive Assessment score of ≤ 23 in the presence of SCD or problems with ADL activities and the absence of DEPR.

| **Retinal microvascular parameters** | **All (n=1431)** | **No MCI (n=1370)** | **MCI (n=61)** | **P-Value** |
| --- | --- | --- | --- | --- |
| Mean CRAE (PX, SD) | 29.643 ± 2.221 | 29.627 ± 2.207 | 29.987 ± 2.523 | 0.22 |
| Mean CRVE (PX, SD) | 40.844 ± 3.268 | 40.862 ± 3.275 | 40.427 ± 3.104 | 0.31 |
| Mean AVR (SD) | 0.729 ± 0.061 | 0.728 ± 0.061 | 0.745 ± 0.073 | 0.04 |
| Mean fractal dimension arteriolar (SD) | 1.557 ± 0.053 | 1.557 ± 0.053 | 1.556 ± 0.045 | 0.86 |
| Mean fractal dimension venular (SD) | 1.540 ± 0.051 | 1.540 ± 0.051 | 1.536 ± 0.048 | 0.58 |
| ^a^Mean tortuosity arteriolar (SD) | 0.114 ± 0.157 | 0.113 ± 0.155 | 0.137 ± 0.208 | 0.25 |
| ^a^Mean tortuosity venular (SD) | 0.067 ± 0.106 | 0.067 ± 0.108 | 0.066 ± 0.065 | 0.93 |

Values are n (%) for categorical variables and mean ± SD for continuous variables. P values were calculated by independent samples t tests. Abbreviations: MCI, mild cognitive impairment; CRAE, central retinal arteriolar equivalent; CRVE, central retinal venular equivalent; AVR, retinal arteriole/venular ratio; SD, standard deviation; PX, Pixels. ^a^Tortuosity values were multiplied by 1000 in order to be shown in table. P <0.05 was considered statistically significant.

**Supplementary Table 3**. Logistic regression analysis of retinal microvascular parameters and Mild Cognitive Impairment status characterised by a Montreal Cognitive Assessment score of ≤ 23 in the presence of SCD or problems with ADL activities and the absence of DEPR.

|  | **Minimally Adjusted** | | | | **Fully Adjusted** | | |
| --- | --- | --- | --- | --- | --- | --- | --- |
| **Retinal parameter** | **OR** | **95% CI** | **P-Value** | **OR** | | **95% CI** | **P-Value** |
| ^a^CRAE (PX) | 1.15 | 0.89, 1.47 | 0.29 | 1.10 | | 0.85, 1.43 | 0.48 |
| ^a^CRVE (PX) | 0.85 | 0.66, 1.11 | 0.24 | 0.89 | | 0.68, 1.18 | 0.44 |
| ^a^AVR | 1.30 | 1.02, 1.67 | 0.04 | 1.22 | | 0.93, 1.60 | 0.15 |
| ^a^Fractal dimension arteriolar | 1.01 | 0.78, 1.31 | 0.93 | 0.93 | | 0.72, 1.21 | 0.60 |
| ^a^Fractal dimension venular | 0.96 | 0.74, 1.24 | 0.75 | 0.90 | | 0.69, 1.18 | 0.45 |
| ^ab^Tortuosity arteriolar | 1.09 | 0.84, 1.40 | 0.53 | 1.08 | | 0.83, 1.40 | 0.59 |
| ^ab^Tortuosity venular | 1.03 | 0.80, 1.33 | 0.84 | 1.05 | | 0.81, 1.35 | 0.74 |

Abbreviations: CRVE, central retinal venular equivalent; AVR, retinal arteriole/venular ratio; CI, confidence interval; OR, odds ratio; PX, pixels. ^a^RMPs were transformed into standardised AZ-scores before inclusion in regression models. ^b^Tortuosity values were skewed and therefore log-transformed before inclusion in regression models. Minimally adjusted model: age and sex. Fully adjusted model: age, sex, alcohol consumption, smoking status, educational attainment, physical activity, history of cardiovascular disease, hypertension, triglycerides, diabetes, medication, mean arterial blood pressure, body mass index and high density lipoprotein. P <0.05 was considered statistically significant.

**Supplementary Table 4**: Comparison of demographic characteristics between all participants with retinal fundus imaging with and without VAMPIRE retinal measures.

| **Participant characteristics** | **Participants with images included (n=1431)** | **Participants with images excluded (n=1117)** | **P value** |
| --- | --- | --- | --- |
| Mean age (years, SD) | 62.4 ± 8.5 | 66.3 ± 9.2 | <0.01 |
| Female, n (%) | 750 (52.4) | 552 (49.4) | 0.13 |
| Smoking status, yes n (%) | 115 (8.0) | 115 (10.3) | 0.05 |
| Alcohol consumption, non-drinker, n (%) | 299 (20.9) | 297 (26.6) | <0.01 |
| Education, secondary level and above, n (%) | 1251(87.4) | 931 (83.3) | <0.01 |
| Diabetes, yes n (%) | 308 (21.5) | 154 (13.8) | <0.01 |
| Mean BMI (kg/m^2^, SD) | 28.2 ± 4.6 | 28.9 ± 5.0 | <0.01 |
| Mean arterial blood pressure (mmHg, SD) | 98.1 ± 12.6 | 104.4± 49.2 | <0.01 |
| Cardiovascular disease, yes n (%) | 90 (6.3) | 128 (11.5) | <0.01 |
| Hypertension, yes n (%) | 432 (30.2) | 442 (39.6) | <0.01 |
| Mean triglyceride (mmol/L, SD) | 1.6 ± 0.9 | 1.6 ± 0.8 | 0.41 |
| Mean HDL cholesterol (mmol/L, SD) | 1.6 ± 0.5 | 1.6 ± 0.4 | 0.01 |
| Mean MoCA test score (SD) | 26.0 ± 2.8 | 25.2 ± 3.2 | <0.01 |

Values are n (%) for categorical variables and mean ± SD for continuous variables. P values were calculated by independent samples t and chi squared tests. Abbreviations: BMI, body mass index; HDL, high-density lipoprotein; MoCA, Montreal Cognitive Assessment; SD, standard deviation. P < 0.05 was considered statistically significant.
